# What understanding of economics do medical students have?

**DOI:** 10.3205/zma001249

**Published:** 2019-08-15

**Authors:** Anke Spura, Katrin Werwick, Bernt-Peter Robra, Christoph Stallmann, Stefanie March, Nadine Ladebeck, Rüdiger Braun-Dullaeus, Philipp Stieger

**Affiliations:** 1 Federal Centre for Health Education, Unit 2-24, continuiing education/qualification/university cooperation, Cologne, Germany; 2 Otto-von-Guericke-University Magdeburg, Institute of Social Medicine and Health Economics, Magdeburg, Germany; 3 Otto-von-Guericke-University Magdeburg, Medical Faculty, Office of the study dean, Magdeburg, Germany; 4 Hochschule Magdeburg-Stendal, FB Soziale Arbeit, Gesundheit und Medien, Magdeburg, Germany; 5 University hospital Magdeburg, university clinic of Cardiology and Angiology, Magdeburg, Germany

**Keywords:** health economics, economization, medical education, medical students, qualitative research

## Abstract

**Introduction: **Economic topics appear in the medical studies curriculum at different times. Despite socio-political relevance, there is hardly any information about the degree of understanding that medical students have of “economics in medicine”. The present study addresses the questions: What understanding of “economics in medicine” do medical students have before the start of the Practical Year? To what extent is economic teaching content understood as “economization” from outside the profession?

**Method: **Magdeburg medical students in the 5^th^ year of study, who participated in preparatory seminars for the Practical Year (PY) in 2014 and 2015 (60 participants each), assessed the relevance of various seminar topics four months prior to the start of the semester.

On the basis of a three-stage qualitative-reconstructive partial evaluation, students’ economic understanding is explored through secondary analysis:

1. deductive derivation of the analysis units;
2. integrative basic method (“segmentation”, “micro-linguistic detailed analysis”, “central theme”);
3. development of a theoretical model by placing the central themes in context following Grounded Theory.

**Results: **Based on the theory, 19 free-text answers with economic reference were identified from the total of all free-text answers. Each answer was assigned to at least one of a total of six themes of the students’ understanding of economics:

1. de-professionalizing economization,
2. deciding and working economically,
3. ambivalent requirements for efficiency and equity,
4. the doctor as an entrepreneur,
5. economics as relevant learning content,
6. PY as a conflict-laden setting for economized working and learning.

The theoretical model contains social, praxeological and professional references, which can themselves be ambivalent and conflicting.

**Conclusion: **Despite their critical attitude, the surveyed medical students are neither hostile to economics nor do they regard economics in medicine as a taboo subject.

Economic learning content is recognized as relevant. Educational formats that tackle the tension between patient and system orientation in a problem-oriented manner can be a productive setting for economic reflection.

## Zusammenfassung

**Einleitung: **Ökonomische Themen sind im Medizinstudium zu verschiedenen Zeitpunkten curricular verankert. Trotz gesellschaftspolitischer Relevanz bestehen kaum Kenntnisse darüber, welches Verständnis Medizinstudierende von ‚Ökonomie in der Medizin‘ haben. Die vorliegende Untersuchung geht den Fragen nach: Welches Verständnis von ‚Ökonomie in der Medizin‘ haben Medizinstudierende vor dem Beginn des Praktischen Jahres? Inwiefern werden ökonomische Lerninhalte als professionsfremde „Ökonomisierung“ verstanden?

**Methode: **Magdeburger Medizinstudierende im 5. Studienjahr, die 2014 und 2015 an Vorbereitungsseminaren für das Praktische Jahr (PJ) teilnahmen (je 60 Teilnehmende), schätzten vier Monate vor Seminarbeginn teilstandardisiert-fragebogengestützt die Relevanz verschiedener Seminarinhalte ein.

Anhand einer dreistufigen qualitativ-rekonstruktiven Teilauswertung werden studentische Ökonomieverständnisse sekundäranalytisch exploriert:

1. Deduktive Ableitung der Analyseeinheiten;
2. Integratives Basisverfahren („Segmentierung“, „mikrosprachliche Feinanalyse“, „zentrales Motiv“);
3. Inbezugsetzung der zentralen Motive als theoretisches Modell nach Grounded Theory.

**Ergebnisse: **19 Freitextantworten mit ökonomischem Bezug wurden theoriegeleitet aus dem Gesamt aller Freitextantworten identifiziert. Jeder Antwort wurde mindestens eine von insgesamt sechs Motiven studentischen Ökonomieverständnisses zugeordnet:

1. De-professionalisierende Ökonomisierung,
2. Wirtschaftlich entscheiden und arbeiten,
3. Ambivalente Effizienz- und Gerechtigkeitsanforderungen,
4. ÄrztIn als UnternehmerIn,
5. Ökonomie als relevanter Lerninhalt,
6. PJ als konflikthaftes ökonomisiertes Arbeits- und Lernsetting.

Das theoretische Modell beinhaltet gesellschaftliche, praxeologische und professionelle Bezüge, die in sich ambivalent und zueinander konflikthaft sein können.

**Schlussfolgerung: **Die befragten Medizinstudierenden sind trotz kritischer Haltung weder pauschal ökonomiefeindlich noch tabuisieren sie Ökonomie in der Medizin.

Ökonomische Lerninhalte sind als relevant anerkannt. Lehrformate, die problemorientiert das Spannungsfeld zwischen Patienten- und Systemorientierung aufgreifen, können ein produktives Setting für ökonomische Reflexion sein.

## 1. Introduction

On the basis of expediency, effectiveness but also economic efficiency, Paragraph 70 of Volume V of the German Social Code gives a legal mandate for a needs-based health care system. Debates about what needs should legitimately be met occupy both the boards of independently administered organizations but the issue is also often contentious in everyday medical practice [1]. Therefore, the relationship between economics and medicine is not just topic for specialists of health economics. It is observed and commented upon – at times in a way critical of economics – by a broad public audience represented by various interest groups [2], [3], [4], [5], [6], [7], [8], [9], [10], [11], [12], [13]. For instance according its strategy paper, the Association of the Scientific Medical Societies in Germany (AWMF) sees “patient-oriented, evidence-based medicine, the well-being of the patient and equitable supply” in danger [14]. The decisions of a doctor are implied to be decisions on allocation and always bind resources themselves, for example when taking a patient’s history, when communicating test results and patient information or in interdisciplinary and interprofessional case discussions [14]. 

This means that core medical activities are economically relevant and simultaneously touch upon multiple medical competences. The acquisition of skills begins during medical studies.

In order to improve interdisciplinary education, the 2002 amendment to the Medical Licensure Act [http://www.gesetze-im-internet.de/_appro_2002/index.html] introduced cross-sectional areas into the curriculum of the second phase of studies, including cross-sectional area Q3 “Health Economics, The Health Care System, Public Health” [15]. However, economic subjects in medical studies already appear in the first phase of studies under the subject of “medical sociology” [16]. Likewise, they can be found in the National Competence-Based Catalog of Learning Objectives (NKLM) [http://www.nklm.de] across all disciplines up to the competences for the Practical Year. (Details see attachment 1 .)

The discourse on economic decisions in medicine is also followed closely by the media. Surprisingly, one of the central topics of the health care system is hardly mentioned in educational research. The topic experienced a short boom with the introduction of Q3. A questionnaire-based survey of medical students at three campuses prior to the introduction of Q3 found that the level of knowledge about the health care system and its economic realities was “poor” and that the curriculum did not adequately prepare for professional medical life on this point [17]. This was followed by some Q3 descriptions of teaching concepts [18], [19], [20], [21], [22]. One publication noted a temporal but not causal relationship between Q3 participation and knowledge increase [23]. Nonetheless, continuing education authorities assessed the skills of young professionals, “to take into account economic aspects, especially in the indication for technical examinations [...], as poor” [24]. In turn, a survey of clinicians in training shows that the medical managerial role, which also includes economic skills in the sense of the CanMEDS role profiles, is least relevant for both their own work as doctors and training during the Practical Year [25]. This aligns with a student survey [26], which classifies the socio-economic subjects in the second phase of studies as least important, although all 747 respondents felt that they were inadequately trained in those subjects.

Nevertheless, it remains unexplored what understanding medical students themselves have. The empirical investigation of this question provides insights into the attitudes of medical students who are about to begin the Practical Year and towards the specific processes of patient care. It can also be the starting point for identifying learning needs, as successful teaching is characterized, amongst other things, by target group orientation. The present study addresses the questions: What understanding do medical students have of “economics in medicine” before the start of the Practical Year (PY)? To what extent are economic learning contents understood as “economization” from outside the profession? [27], [28] (For the concept of economy, see table 1 (Tab. 1)). 

## 2. Method

Medical students in the 5th year of study at the medical faculty in Magdeburg (about 210 per year) who participated in the optional PY preparatory seminar “Fit for PY” in 2014 and 2015 (60 participants each) evaluated the relevance of different seminar contents in a questionnaire-based, partially standardized survey four months before the start of the seminar. In 2014, 47 students (78%) and in 2015, 26 students (47%) participated in the survey.

The questionnaire collected the relevance assessments anonymously using Likert scales with supplementary free-text fields and open questions (detailed seminar description and evaluation [29]). Economic learning content on the “cost-benefit assessment” was rated as least relevant in 2014. This led to the above-mentioned research questions in the follow-up survey in 2015 in order to shed more light on this point. The free-text answers with economic relevance follow three lines of inquiry:

- Q1: How important would you the following learning content in a preparatory seminar for the PY be for you? [Likert Scale] Other. [Free Text]
- Q2: What would you expect from an “ideal” PY? [Free Text]
- Q3: What are – in your opinion – health economic aspects in medical practice? Please name as many aspects as you like in the form of bullet points. [Free Text] (only in 2015)

The three-stage evaluation presented here captures the students’ understanding of economics as a knowledge baseline and attribution of relevance. It therefore requires a qualitative-reconstructive approach. This is particularly suitable for the exploration of research desiderata [30]. 

At the first stage of analysis, based on the theory it was possible to deductively derive 19 units of analysis from the total of all free-text answers in the form of quotes with economic reference. The heuristic category key documents the derivation criteria (see table 1 (Tab. 1)). 

The identified quotes (see attachment 2 ) were evaluated in the second stage according to the integrative basic method [31], which through the steps of “segmentation”, “micro-linguistic detailed analysis” (pragmatic, syntactic and semantic analysis), “central theme” combines linguistic and knowledge-sociological methods. In the analysis process it was possible to assign every quote to at least one topic inductively. 

**Figure 1:**
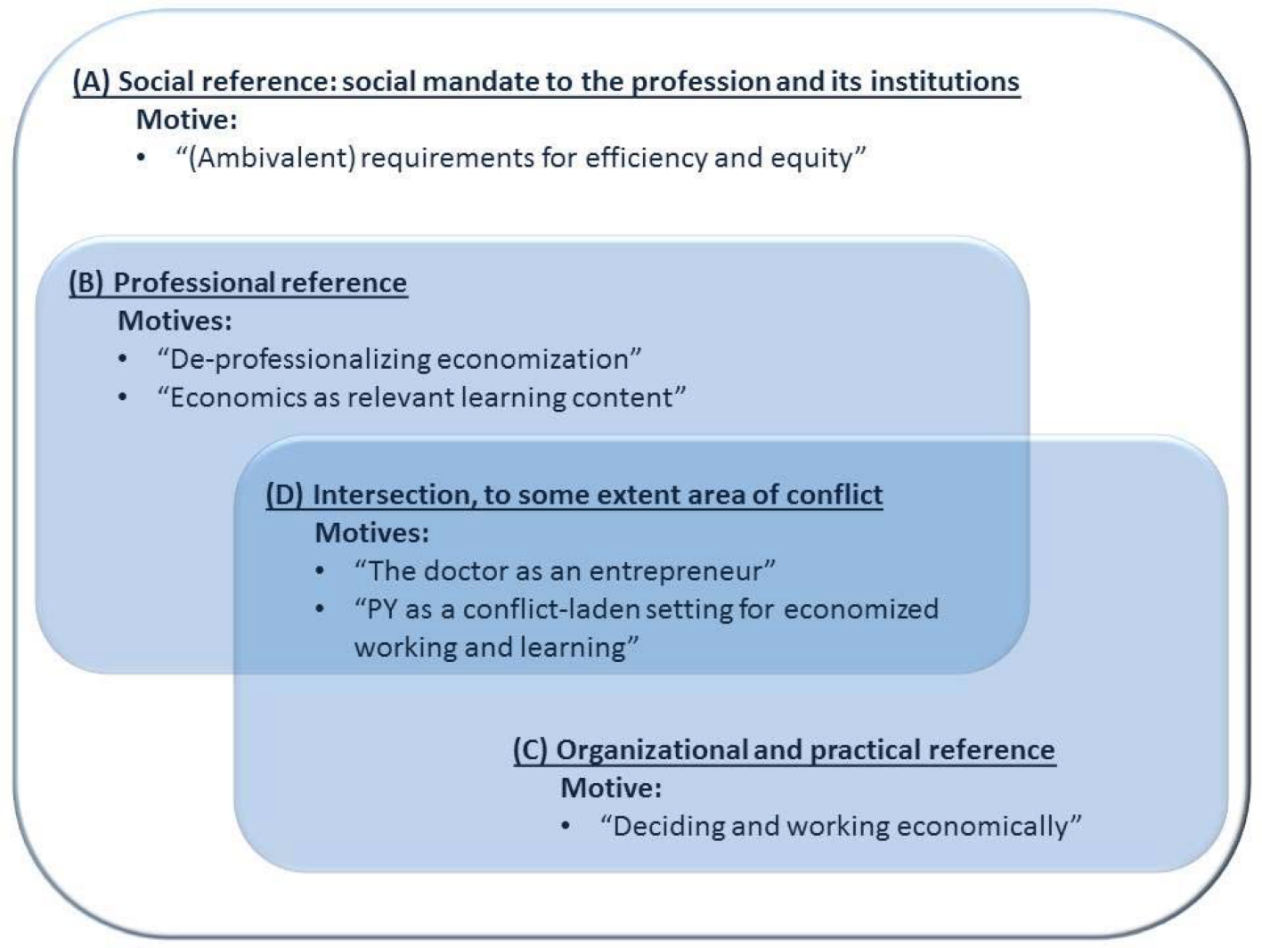
References of the central themes

The third stage consisted of theory generation, in which the central themes were visualized as a diagram in the tradition of Grounded Theory [30], [32] (see figure 1 (Fig. 1)). 

The results were reviewed through discussion by an eight-member interdisciplinary research and author group (sociology, German philology, medicine, public health, MME) in a research workshop [33] on inter-subjective traceability [34] and coherence. 

## 3. Results

19 quotes with economic relevance were identified as analysis units. The results of the detailed micro-linguistic analysis of the integrative basic procedure, focusing on the students’ understanding of economics in medicine (see attachment 2 ) and the theoretical model, are presented by theme and in a condensed fashion.

### 3.1. De-professionalizing economization

Quotes 1 and 4, which form the basis of this category, express a systemic critique of a supposedly advancing economization of medicine, which also penetrates into the core sphere of medical-professional evaluations and decision-making.

Thus, Quote 1 raises the learning content of “cost-benefit balance” as an issue. Central to this is a systemic critique of a supposedly progressive erroneous trend into an economized model of medicine: The student rhetorically doubts the seriousness of the question and justifies her irritation with supposed basic requirements for students. According to these, the following learning goals for young professionals have to be prioritized:

1. *“to find your way into clinical routine”,*
2. *“to learn basic care”.*

In contrast “cost-benefit considerations” go over the head of even experienced physicians and these are therefore out of place in the training curriculum. The medical metaphor of “listing” diagnoses a misalignment or wrong development of the *“medical care system”*, with “*cost-benefit-orientations”* being symptomatic of this. *“Education”* as the functional area, which serves system reproduction through training and socialization is also affected by this systemic misalignment/dysfunction. The wording *“have even already arrived [...]”* indicates a spatial-temporal-oriented progression. Thus, any form of resistance is hindered or prevented at the earliest stage. This phenomenon of economization as a *“taking over”* [27] of a professional core domain is described not as an isolated case but *“time and again”*. In contrast, three basic key competencies of patient care are dismissively ticked off. While the expression “no chance” expresses a negative result of an intended target [https://www.dwds.de/wb/Fehlanzeige], i.e. that taking a *“patient’s history”* still represents a missed target of a nonetheless medical act, the remaining competences (*“clinical examination”, “basic resuscitation”*) are characterized ironically and sarcastically as irrelevant (*“not so important”*) and expendable (*“superfluous”*). This discrepancy between good medical education and practice on the one hand and on the other a – supposedly misguided – curriculum discussion on training content, comes to a head in an economized logic of action: *“But as long as you’re able to weigh cost v benefit”*.

The answer in Quote 4 spans a discursive field between weighing medical (risk-benefit) and economic (cost-benefit) items. She thus represents an aspect in the conflict area of Theme 3.2. The indication of treatment under an economic premise as a core medical task moves between “*damage”* and* “benefit”* in terms of weighing the risk. However, economic arguments dominate the medical if only a vague benefit is foreseeable and the risk assessment remains at a low level (*“can’t hurt”*). The new weighing/calculation therefore moves between two cost-benefit poles, whereas the risk turns out to be a financial one.

In practice this weighing up, permeated with managerial and economical aspects and crucial for decision making about further medical treatment, is negotiated interactively in asymmetrical working relationships and must be legitimized in discourse. However, the negotiation outcome is not a consensual decision as a shared situation-problem-solution strategy but an instruction (*“have to let them tell me”*). Understanding as a learning prerequisite is neither demanded nor expected. Rather, it turns out that medical studies socialize students into hierarchical structures [35].

#### 3.2. Deciding and working economically

Despite criticism of the system, it is acknowledged in the student contributions (Quotes 2-3) that under an imperative of economic efficiency, medical practice must also be economic. 

In their future professional activity both interviewees expect, substantiated by *“decisions”* and* “working”*, that this, as becomes clear in the second quote, will be a constant (*“every day”*) and unavoidable (*“have to”*) need for working economically (in line also with §70 German Social Code V). The author of Quote 2 identifies himself with the task of* “making economic decisions”* as part of a medical collective (*“As doctors”, “we”*). A causal theory on the phenomenon of *“deficient economic working”* is developed and a differentiation is made between doctors who more or less fulfill the economic imperative. Indirectly, a faulty teaching-learning relationship between educators and students becomes apparent. In this case, the disregard of an economic work logic is due to the still incomplete training and socialization process, i.e. missing didactic structures (*“subject”, “taught systematically”*) and faulty role model learning (*“we don’t learn that much in our day to day work”*).

It becomes clear that in practice the normative requirement (*“must”*) is not respected by all (*“that later some doctors work very economically and others less so”*). The disregard for the economic efficiency imperative is said to stem from the fact that this topic is not addressed didactically in training. Decision-making and work processes are therefore said be uncertainly structured from an economic point of view, might succeed or end up being inadequate.

#### 3.3. (Ambivalent) requirements for efficiency and equity

The student responses allow a reconstruction of system-oriented concepts about the social mission of the health care services as normatively formulated system requirements (Quote 5), as demographically derived supply contracts (Quote 6) and as demands for balancing between resource scarcity and an increase in morbidity (Quote 7). 

For instance Quote 5 expresses an expectation of a *“health care system based on solidarity”* that is community-oriented and committed to the protection of vulnerable groups. The entailed (*“low threshold but efficient”*) mode of action is *“work”* in the sense of targeted and purposeful service provision. A low threshold as socially barrier-free access to the health care system is limited by the efficiency imperative, i.e. the obligation to maximize effectiveness while minimizing the use of resources. Although the health economics line of reasoning certainly recognizes the efficiency line of reasoning as a marker of solidarity, the logics of openness and demarcation here stand in opposition, linguistically recognizable in the adversative conjunction *“but”*. Health care is therefore limited economically.

The linguistic comparison of Quotes 5-6 shows border metaphors: explicitly in the expression *“low threshold”* to mark a health care system whose *“older and sicker”* clients will have to depend on solidarity as a mechanism of inclusion [36]. In contrast, the agent *“economization”* stands as a powerful (*“stronger”, “pervasive”*) process. Economization can thus lead to shrinking solidarity, promoting a health care system that is not aimed at its clients.

Also in Quote 7, rising patient numbers and morbidity are expected and associated with increasing scarcity of resources. The medical care mission is seen as less as improved medical and curative care but more as resource distribution which should be as *“fair as possible”*, such as medical human resources, treatments, drugs, cost-intensive technologies. The author acknowledges this mission by formulating the desire for the required knowledge and high competence (*“master”*). Accordingly, doctors are seen to have a powerful legally and morally coded, public welfare-oriented mediation function and are entitled to address care needs and demands despite or due to scarcity of resources.

In this context Quote 6, with a view to the common good, refers to the demographic problem in Quote 7, which states that morbidity and the aging of society are progressing (comparative* “older and sicker”*). The image of the future medical care contract gains momentum and strength by *“economization”* breaking the boundaries of medicine and by its impact being fed (*“bigger and bigger impact”*) through the process of a growing population of people requiring care – represented linguistically by the German proportional conjunction *“je”*. 

#### 3.4. The doctor as an entrepreneur

Career planning and competences beyond those of being a *“medical expert”* [http://www.nklm.de] only become relevant around the time of graduation. In contrast to hospitals where work-sharing and a hierarchical organization are the norm, *“young”* doctors do not commonly see themselves confronted with issues of economics but respondents who are aiming to set up a practice recognize this as being more relevant, as can be seen in Quotes 7 and 8. 

These are willing to assume entrepreneurial responsibility in the form of managers [http://www.nklm.de]. Responsibility, however, also entails economic competence – an economically operating medical practice (“Then* I’ll be the boss and must make sure that the business is running”*), providing health care to the population (*“increasing number of patients and morbidity”*) under conditions of scarcity in the health care system – and at the same time the ethics of allocation (*“crucial to know things about their (as fair as possible) distribution... if you don’t want to be a wanker”*). 

Economic competence and ethical responsibility are said to show in the professional double mission through granting scarce medical resources (for example in the indication of treatment, certificates of incapacity for work) with increasing numbers of patients and morbidity.

#### 3.5. Economics as relevant learning content

Despite the need for economic training content especially for the PY and for medical studies in general, an awareness of the tension between economization and the principle of economic efficiency is expressed. There is also a critical attitude towards the current education system, which does not facilitate the transfer into practical medical contexts. 

For the PY, the students (Quotes 9 and 10) would like to have knowledge of practice management (*“paperwork”*), the* “accounting system”* and economic decision-making logic (*“cost-benefit thinking”*). It turns out that they do wish to acquire concrete procedures and action-guiding logic in the PY that go beyond purely clinical and medical topics. However, a didactic barrier for economic *“theory-practice transfer”* is identified in Quotes 4 and 11. They express a need for understanding how assessment, weighing and decision-making processes (*“assessment”, “how an assessor estimates the value of a doctor’s surgery”*) work through medical care processes and facilities (*“tests or lab results”, “value of a doctor’s surgery”*). There are calls for an integrated curriculum for clinical processes and economic relationships while at the same time reiterating medical self-conception (*“I don’t want a degree in business administration”, “reduce macroeconomics in favor of a crash course in business administration”*).

The respondents rate economic content as useful in these remarks but are critically sensitive about potential curricular economization and current subject-related training practice, as indicated in Quote 12. Despite being part of the curriculum (*“Göko”* = health economics, Q3), the subject is called* “rubbish”* i.e. referred to as stupid, meaningless chatter [28]. A transfer to everyday practice is not recognized in this perception of students, course content on health economics in the subject seem uncoupled from the* “future economics”* of medical practice.

#### 3.6. PY as a conflict-laden setting for economized working and learning

The participants were asked what they expect “*from an “ideal” PY”*. Most answers with economic content relate to the PY and its economics as a conflict-laden working and learning setting both in terms of the roles of students as members of the workforce and as learners. Critical remarks about the PY as a working assignment with no financial recognition of the work done in the PY predominate. 

The students see their input as qualified labor but feel they are used on the wards as assistants (Quotes 13-18).

Quote 19 points to the economic aspect of the PY as a poor learning setting. This shortcoming appears as a daily role conflict between health care and medical education.

#### 3.7. Theoretical model: References of the central themes 

The analysis shows a differentiated perspective of the problem area *“economics in medicine”*. Students certainly consider economic learning content as relevant to medical education but the theoretical curriculum and practical training do not appear linked. 

The reconstructed central themes include social, praxeological and professional references (A-C, see figure 1 (Fig. 1)), which can be ambivalent in themselves and need not be relationally free of conflict.

Students expect a working environment characterized by economic aspects. They are neither opposed to economics in general nor feel that this topic is off-limits. Nevertheless, the reconstruction reveals an over-arching social mission (A) as a paradoxical link between the processes *“demographic aging”* and *“economization of medicine”*: the higher the societal demand for solidarity, the more dominant becomes the economic distribution imperative, with its exclusionary effect. But working economically is not automatically equated with the suppression of *“traditional role expectations of doctors”* [37], (B) allocation decisions in the context of organized medicine, (C) are accepted. Nevertheless from the students’ perspective the relationship between economics and medicine is not tension-free. (D) It is indeed possible to reconstruct a critical attitude towards a profit-oriented medical practice which is not aimed at the patient, especially if this threatens to conflict with *“good”* patient care. 

Now and tomorrow, doctors will have to seek a balance between the professional demands of the patient and the system. Critical attitudes are also recognizable in terms of the PY in its inherent economic concerns, if a conflict of recognition is anticipated: As learners in their PY, they expect high-quality training from their training organization – *“in exchange”* for their *“work input”* in patient care. However, they already anticipate that this exchange will not always be fulfilled. This expectation of a gratification crisis [38] comes to a head as a result of inadequate pay for work as *“student labor”*.

## 4. Discussion and conclusions

This analysis reconstructs the student perspective and the status of their health economics concepts after completion of the theoretical university education and shortly before the PY and increasing practical responsibilities in patient care. It explores initial answers to a research desideratum and provides an empirical contribution to the discourse on economization and its possibilities for mediation in medicine. The students’ comments already show a multifaceted spectrum of themes. They reflect the tensions between economization, equity of supply and quality orientation. 

The integrative basic method [31] was used for the first time in the context of medical didactic research and proved to be productive for an exploratory approach. The informative value of this exploratory study, however, is based on a small group of students who may be more attuned to the topic. The *“Fit for PY”* context may have caused a selection of medical students who are particularly interested in new and improved training formats. A control group outside those preparing for the PY was not interviewed. 

In the sense of contrastive comparison, the limited data also shows no maximum contrast, for example in the form of an appreciative attitude toward economization tendencies. It would be worthwhile to expand this first exploration through further qualitative research in the sense of theoretical sampling and for theoretical saturation. It is therefore not possible to make statistically representative statements about medical students in Magdeburg or beyond. This would require a more differentiated system-related health economic and patient-oriented socio-economic operationalization which be presented to a broader sample and in contrasting locations in order to contribute to evidence-based and target-group-oriented training (research) [39], [40], [41]. 

Nevertheless, there are implications for the teaching of economic learning content. One task is to link abstract economic knowledge with practical patient-related decision making and care processes. This corresponds to one aspect of the study by Dafsari et al. [26], according to which in particular students with a preference for General Practice would like more instruction in health economics but in a way that focuses on issues of practical management (such as accounting structures). There is also a need for offers that allow students to reflect on the areas of tension. One possibility would be a curricular framing of the PY, by placing student expectations and experiences, amongst other things, into an economic context and linking them didactically with the subject content, e.g. from medical sociology [42], Q3 Health Economics but also medical ethics (see attachment 1 ). By spanning the curriculum in this manner, learning content from the first and second phase of medical studies could be integrated. For medical internships such as the PY, it would be helpful to sensitize medical lecturers to this challenging topic. Problem-oriented case discussions using either case histories and typified case vignettes [43], as implemented in the *“Fit for PY”* preparatory seminars [29], or on the basis of PY logbooks, could be useful approaches. They would offer future physicians an opportunity to learn to develop and represent their role in the health care system, in real-life patient care and also in the debate on economics.

## Ethics Committee vote

The ethical standards of project implementation and evaluation have been adhered to (positive ethics vote by the Ethics Committee of the Medical Faculty, Otto von Guericke University Magdeburg, No. 65/15).

## Acknowledgements

The authors would like to thank the “Fit for PY” participants and the members of the qualitative research workshop of the Institute for Social Medicine and Health Economics at the Medical Faculty of Magdeburg.

## Competing interests

The authors declare that they have no competing interests. 

## Supplementary Material

Overview Economic Learning Content in Medical Studies (IMPP Subject Catalogs, Content Catalogs, National Competence-Based Catalogue of Learning Objectives (NKLM))

Numbered quotes with economic reference with assigned theme numbers (in line with to Chapter 3)

## Figures and Tables

**Table 1:**
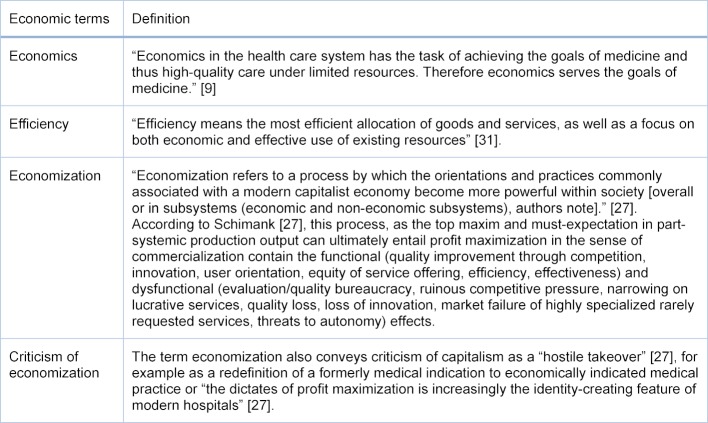
Heuristic category key for the deductive identification of the analysis units
