# Supplementary material for: What understanding of economics do medical students have?
Source: GMS J Med Educ. 2019 Aug 15;36(4):Doc41. doi: 10.3205/zma001249 (PMC6737257; doi:10.3205/zma001249)
Supplement: Overview Economic Learning Content in Medical Studies (IMPP Subject Catalogs, Content Catalogs, National Competence-Based Catalogue of Learning Objectives (NKLM)) [file JME-36-4-41-s-001.pdf]

**1. Phase of medical studies: IMPP Subject Catalog 1 [1]**	
Sub-catalog “Basics of Medical Psychology and Medical Sociology”	Influences of social opportunity structure (**social stratification**, social networks, **labor market**, education); social capital; **income inequality; social cohesion; structural deprivation, influences of economic** and ecological **environmental factors (industrialization, urbanization, globalization)**
	Methodological basics, test diagnostics: [...] **economy** [...]
	Organizational and **economic determinants of medical practice; Importance of economy and expediency**
	Ethical and **economic problems of prevention**
**2. Phase of medical studies: Cross-sectional areas, social medicine [2,33]**	
Q1 “Epidemiology, medical biometry, medical IT”	Evaluation of clinical, diagnostic, therapeutic, prognostic and **health economic studies**
	Main findings of disease-related epidemiology: [...] **health economic considerations for diagnostics** [...]
Q2 “History, theory, medical ethics”	health care:<br>– [...] criteria for fair health care provision (equal opportunities, needs, benefits, **efficiency, merit**)<br>– Prioritization and **rationing in health care**; ethical analysis of the **health economic conditions; levels of allocation (micro and macro allocation)**
Q3 “Health economics, health care system, public health care”	**Health economics** *(complete)*: **Basic concepts and relationships, financing of the health care system, types of studies of health economic, cost effectiveness,** international comparison of health care systems, **control problems and approaches**
	Health care system: [...] **developments in the area of financing, remuneration, performance, service catalog/“self-responsibility”/special regulations, service requirements**
Q10 “Prevention, health promotion”	Relevance of prevention and health promotion [...], **costs in the health care system**, [...]; **structure and potential messages of cost-benefit analyzes (cost [cost] analyzes [e.g. medical expenses analysis], cost-effectiveness analyzes [cost-minimization, cost-utility analyzes], cost-benefit analyzes)**
Q12 “Rehabilitation, physical medicine, naturopathy”	– Rehabilitation goals: [...] also from an **economic point of view** for society<br>– Socio-medical assessment: **Definitions of incapacity to work, capacity in working life** with positive and negative performance levels in contrast to the additional consideration of socio-legal aspects with partial and full **reduction of earning capacity** (MdE)<br>– Initiation (assignment) and guidance of the rehabilitation process: Definition and implementation of the assignment with consultation of the patient, the treating physicians, other service providers and **funding agencies**<br>– **Financing** and service providers of rehabilitation
Social medicine	– **Social** and medical determinants of health and disease,<br>– **Social** aspects of health and disease<br>– **Social security** for defined life risks
**NKLM: Fields of competence and chapters with declared economic “PY competence” (content description in NKLM learning objective/subject relevance/milestone) [3]**	
9 “The doctor as health adviser and advocate”	explain medical, educational, normative-regulatory and **socioeconomic impacts** on individuals’ health and healthy lifestyles.
10 “The physician as bearer of responsibility and manager”	They master **efficient and effective medical work**, demonstrate and define problems and work out solutions.

	– recognize and unpick medical, social, cultural, age- and gender-related issues in specific patients with acute powerlessness, if appropriate **identify the economic conflict** [...]<br>– **identify** a specific health care situation using the **DRG system** and assess the consequences of their medical decisions for **billing**.<br>The graduate handles **resource allocation** responsibly. […]<br>– make and justify statements in a given medical patient situation about the decisions **“(in)ability to work”, “occupational disability” and “partial, complete or temporary, permanent reduction in earning capacity”**.<br>– [...] statements on decisions to apply for recognition of the **“degree of disability”** [...].<br>– [...] statements about decisions to apply for **“care levels”** [...].<br>– [...] **methods of resource allocation** [...]. They can understand and explain instruments **for resource allocation in medical practice** as well as resulting conflicts.
14c “Guiding a medical conversation”	explain health policy and **economic factors** influencing decision-making [...]
	have a differentiated perception of cultural, social, gender, **socio-economic**, religious and ideological value and norm systems in themselves and others
19 “Health promotion and prevention”	adapt counseling sessions to the gender, cultural and **socio-economic background** of patients.
	describe the **health economic relevance** and legal targets on prevention and health promotion.
	identify relevant epidemiological data and registers and explain their importance for preventive medical and **economic health measures**
