# Supplementary material for: What understanding of economics do medical students have?
Source: GMS J Med Educ. 2019 Aug 15;36(4):Doc41. doi: 10.3205/zma001249 (PMC6737257; doi:10.3205/zma001249)
Supplement: Numbered quotes with economic reference with assigned theme numbers (in line with to Chapter 3) [file JME-36-4-41-s-002.pdf]

Quote No.	Quote content	Query stem	Theme No.
1	“Cost-benefit considerations? Seriously? As a student you should first find your way into clinical routine and learn basic care before dealing with problems that often go over the head of even experienced doctors. This once again shows that the list of our health care system has even made it into the education system. Competent at doing patient history - no chance. Clinical examination - not so important. Basic resuscitation - superfluous. “But as long as you’re able to weigh cost v benefit...” (2014)	Q1	3.1
2	“As doctors, we have to make economic decisions every day” (2015)	Q3	3.2
3	“In medical studies there’s no separate subject in which the basics of working economically are explained and taught systematically. I think that later some doctors work very economically and others less so. It doesn’t become apparent that often, with those doctors who aren’t as good at being economical and as a result we don’t learn that much in our day to day work.” (2015)	Q3	3.2
4	“I don’t want a degree in business administration but personally what I totally lack is an understanding of how expensive tests or lab results are. In some situations, I think an additional test can’t hurt and then I have to let them tell me that it would be too expensive in terms of the benefits that the result would bring.” (2015)	Q3	3.3, 3.6
5	“A health care system based on solidarity should have a low threshold but be efficient.” (2015)	Q3	3.4
6	“The economization of medicine is getting stronger and more pervasive the older and sicker our society becomes, we can and we are experiencing this process ourselves, so preparing for it, no matter where and in what field you’ll be working later on, is so important.” (2015)	Q3	3.4
7	“I want to be aware of and master this, as I may want to set up a practice myself where scarcity of resources with an increasing number of patients and morbidity makes it crucial to know things about their (as fair as possible) distribution. ...if you don’t want to be a wanker...” (2015)	Q3	3.4, 3.5
8	“I want to open up a practice later on. Then I’ll be the boss and must make sure that the business is running” (2015)	Q3	3.5
9	“to get even better insights into how a GP works [...] including “paperwork” (transfers, admissions, accounting systems, and so on), I’d like to know more about that.” (2014)	Q2	3.6
10	“Especially to be familiar with clinical processes, documentation and cost-benefit thinking, who is allowed to do what and which interdisciplinary routes there are and how you follow them.” (2014)	Q2	3.6
11	“The following text refers more to medical studies and can’t be done in the “Fit for PY”. I worry that it might reduce macroeconomics in favor of a crash course in business economics. It is very forward-looking to know how one arrives at recommendations for action or to plan the needs of medical practices. Most important, however, was the fact that they are planned. it would be, for example, interesting to hear how an assessor estimates the value of a doctor’s surgery. Or a realistic cost analysis of a random doctor’s surgery with 3 nurses. (2015).”	Q3	3.6
2	“it’s practically not taught. Instead of doing just rubbish Göko, such things would be much more useful. You’re really not prepared for economics in the future at all.” (2015)	Q3	3.6
13	“Adequate pay (minimum wage), because admission requirements to the PY is an Abitur, a 3 month nursing internship, having passed the oral and written	Q2	3.7

	Intermediate Examination in Medical Studies, a 4 month internship and a successful written State Examination. We are more than just “stupid interns” and so should be appropriately remunerated. The current “hourly wage”, if you can call it that, amounts to €200 for 4 weeks of 40 hours = €1.25. Now, considering that many students have to pay for housing and take care of children, that’s more than ridiculous. I will have to work weekends alongside my PY. Thanks a lot!” (2014)		
14	“More money than you university has the cheek to pay, (minimum wage or more than someone on a Harz IV program, which would be an almost embarrassingly large amount) less than 5-6 hours overtime a day [...] holidays, etc.” (2014)	Q2	3.7
15	“Not only having to run errands and fill in for unpleasant jobs (cheap full-time workers to hold hooks and take bloods).” (2015)	Q	3.7
16	“No offsetting of the PY salary against student loans” (2015)	Q2	3.7
17	“The PY is rather poorly remunerated because it’s considered part of your training period, many fellow students are used as a cheap labor in the PY though and the learning effect is limited by quickly asking too much - or too little - of the students.” (2015)	Q2	3.7
18	“not to be purely used as free help for activities where you don’t learn that much.” (2015)	Q2	3.7
19	“Colleagues who enjoy explaining things to you and who aren’t too rushed off their feet. Good working atmosphere. That you don’t get asked to do things alone that you haven’t fully mastered yet but you get to do them a few times under supervision without getting an earful!!!!!” (2015)	Q2	3.7
